# Compressive SAR Imaging with Joint Sparsity and Local Similarity Exploitation

**DOI:** 10.3390/s150204176

**Published:** 2015-02-12

**Authors:** Fangfang Shen, Guanghui Zhao, Guangming Shi, Weisheng Dong, Chenglong Wang, Yi Niu

**Affiliations:** School of Electronic Engineering, Xidian University, Xi'an 710071, China; E-Mails: ffshen@mail.xidian.edu.cn (F.S.); gmshi@xidian.edu.cn (G.S.); wsdong@mail.xidian.edu.cn (W.D.); clwang1221@gmail.com (C.W.); niuyi@mail.xidian.edu.cn (Y.N.)

**Keywords:** SAR, compressive sensing, adaptive sparse representation, random sensing measurements

## Abstract

Compressive sensing-based synthetic aperture radar (SAR) imaging has shown its superior capability in high-resolution image formation. However, most of those works focus on the scenes that can be sparsely represented in fixed spaces. When dealing with complicated scenes, these fixed spaces lack adaptivity in characterizing varied image contents. To solve this problem, a new compressive sensing-based radar imaging approach with adaptive sparse representation is proposed. Specifically, an autoregressive model is introduced to adaptively exploit the structural sparsity of an image. In addition, similarity among pixels is integrated into the autoregressive model to further promote the capability and thus an adaptive sparse representation facilitated by a weighted autoregressive model is derived. Since the weighted autoregressive model is inherently determined by the unknown image, we propose a joint optimization scheme by iterative SAR imaging and updating of the weighted autoregressive model to solve this problem. Eventually, experimental results demonstrated the validity and generality of the proposed approach.

## 1. Introduction

Due to its day/night and all weather performance capabilities, synthetic aperture radar (SAR) has become one of the most promising remote sensing tools in military and civilian fields, including target recognition, topographic mapping, and environmental monitoring. SAR is capable of producing high-resolution images of stationary surface targets and terrain reflectivity. In general, to achieve high-resolution performance, a wideband transmitted signal as well as a large synthetic aperture size are required. However, such requirements lead to a high sampling rate in both the range and azimuth dimensions, which poses a challenge to the analog-to-digital (AD) converter at the receiver and makes the consequent processing complex.

Based on the assumption that SAR images can be sparsely represented in some spaces, such as wavelet, discrete cosine transform (DCT), or Fourier domain, the recently emerged Compressed Sensing (CS) [1,2] theory demonstrates that the SAR image can be exactly recovered with high probability from very limited measurements by solving a convex optimization. Motivated by this, many works [3–6] have applied the CS theory into the SAR imaging and developed a new compressive SAR imaging approach (CS-SAR for short), in which a series of advantages, such as high resolution and low sidelobes, are provided. In addition, considering that SAR imaging is inevitably disturbed by uncertain phase errors in practice, sparsity-driven approaches [7,8] are proposed for joint SAR imaging and phase error correction. Though the superior capability of the compressive SAR imaging approach has been extensively studied, only simple scenes with isolated scatterers existing in a low-reflectivity surrounding are considered. Previous studies [9,10] were capable of dealing with more general cases and demonstrated that a satisfactory imaging performance can be achieved when a SAR image can be sparsely represented in an elaborately selected space. However, a fixed space lacks adaptivity in characterizing the varying structure of the scene. Especially when the waveforms vary significantly across the whole image, reconstruction with some fixed space may suffer from an unsatisfactory performance.

In this paper, an adaptive sparse representation scheme derived from the weighted autoregressive (AR) model is introduced in compressive SAR imaging. The work is inspired by [11], where the AR model is integrated into the compressive image recovery for exploiting the structural sparsity of natural images. The premise of the work is that the piecewise statistical stationary assumption holds. However, a more general case, which statistically in a local window is non-stationary, is considered in our paper. Specifically, considering the local non-stationary property within the SAR image, the similarity between any two pixels, evaluated via the structural similarity probability (SSP), is incorporated into the AR model to further promote the capability of the structural sparsity exploration. With this weighted AR model, a new approach of compressive SAR imaging with adaptive sparse representation (ASR-CS-SAR for short) is developed. Since the parameters of the weighted AR model are inherently determined by the unknown SAR image, an alternative optimization scheme is introduced to join SAR imaging and parameters estimation. In the end, experimental results demonstrate that the proposed ASR-CS-SAR approach outperforms general CS-SAR approaches with fixed spaces in terms of both visual quality and other metrics.

The remainder of this paper is organized as follows. Section 2 gives a brief review of the spotlight model SAR. The general CS-SAR approach is presented in Section 3 and the proposed ASR-CS-SAR approach is given in Section 4. In Section 5, simulation results are presented to demonstrate the validity of the proposed approach. Finally, conclusions are made in Section 6.

## 2. SAR Imaging Model

In this section, we give a brief review of the spotlight mode SAR. The radar traverses along a straight path with a constant speed, and continuously steers the antenna beam to a fixed ground patch of interest. At each location, radar transmits electromagnetic pulses and records the return signal from the ground patch. Then, the collected SAR echoes from multiple observation locations are processed to image the reflectivity profile. In spotlight mode SAR, the most commonly used pulse is

(1) $$\text{s}\left(t\right)=p\left(t\right)e^{j\omega_{0}t}$$

where *p*(*t*) is the transmitted waveform, *t* represents the signal time, and ω_0_ is the carrier frequency. Usually, chirp signal is adopted as transmitted waveform

(2) $$p\left(t\right)=\mathrm{rect}\left(\frac{t}{T}\right)e^{j\pi \gamma t^{2}}$$

where rect(·) denotes a rectangular window, γ is the chirp rate, and *T* represents the signal duration. Supposing the whole scene consists of a series of point scatterers on a grid, the scattering response can be approximated as a sum of the responses from individual point scatterers. As a consequence, the corresponding echo signal at an aspect angle θ after demodulation can be described as

(3) $${\text{r}}_{\theta }\left(t\right)=\iint_{\left(x,y\right)\in \text{S}}\text{Q}\left(x,y\right)p\left(t-2R\left(\theta ,x,y\right)/c\right)e^{-j2\omega_{0}R\left(\theta ,x,y\right)/c}\text{dxdy}$$

Where S denotes the illuminated scene, ***Q***(*x*,*y*) represents the reflectivity coefficient of the point scatterer at coordinates (*x*,*y*), *R*(θ,*x*,*y*) is the distance between the radar and the point scatterer, and *c* is the speed of light. Note that the SAR image process is usually operated in the discrete domain, which includes two aspects. On the one hand, the illuminated scene is discretized into a *N_a_* × *N_r_* grid. Let ***q*** be a lexicographic ordered vector of an unknown sampled reflectivity image ***Q***(*x*,*y*) of length *N* = *N_a_* × *N_r_*. On the other hand, temporal samplings in both range and azimuth are also discrete. Let ***r***_θ_(*t_s_*) be the *s*-th sample of ***r***_θ_(*t*), we have

(4) $${\text{r}}_{\theta }\left(t_{s}\right)=\sum_{i=1}^{N}{\text{q}}_{i}p\left(t_{s}-2R\left(\theta ,i\right)/c\right)e^{-j2\omega_{0}R\left(\theta ,i\right)/c},s=1,2,\ldots ,S,$$

where *R*(θ,*i*) corresponds to the two-way distance between the radar and the *i*-th point scatterer, and *S* is the sampling number in range dimension. Considering that SAR imaging is normally synthesized via multiple aspect angles and the real echoes are contaminated by the additive Gaussian noise, the practical SAR echoes can be expressed as

(5) $$\begin{array}{ccc} \left[\begin{array}{c} {\text{r}}_{\theta_{1}}\\ {\text{r}}_{\theta_{2}}\\ \ldots \\ {\text{r}}_{\theta_{L}} \end{array}\right]=\left[\begin{array}{c} {\text{C}}_{\theta_{1}}\\ {\text{C}}_{\theta_{2}}\\ \ldots \\ {\text{C}}_{\theta_{L}} \end{array}\right]\text{q}+\varepsilon , & \text{or} & \text{r}=\text{Cq}+\varepsilon , \end{array}$$

Where ***r***=[***r***_θ_1__(*t*_1_),⋯,***r***_θ_1__(*t*_s_),***r***_θ_2__(*t*_1_),⋯,***r***_θ_2__(*t*_s_),⋯,***r***_θ_*L*__(*t*_1_),⋯,***r***_θ_*L*__(*t*_s_)]*^T^* and *L* is the total number of aspect angle. ***C*** serves as the SAR observation matrix with its element ***C***_θ_*l*__=[***C***_θ_*l*__(*t*_1_),***C***_θ_*l*__(*t*_2_),⋯,***C***_θ_*l*__(*t*_s_)]*^T^*, in which ***C***_θ_*l*__(*t*_s_)=[***C***_θ_*l*__(*t*_s_,1),***C***_θ_*l*__(*t*_s_,2),⋯,***C***_θ_*l*__(*t*_s_,*N*)] and ***C***_θ_*l*__(*t*_s_,*i*)=*p*(*t_s_*−2*R*(θ*_l_*,*i*)/*c*)*e*^−^*^j^*^2ω_0_^*^R^*^(θ_1_,^*^i^*^)/^*^c^* **ε** denotes the additive noise. The procedure to obtain the unknown reflectivity ***q*** from the echo ***r*** is referred to as SAR imaging [12].

## 3. CS-SAR Approach

In this section, a brief review of CS theory is given, and then both an efficient undersampling scheme and the mathematical formation of the CS-SAR approach are presented.

### 3.1. Review of CS Theory

Suppose a signal **x** of size *N* × 1 is *K*-sparse in a basis **Ψ**, then it can be described as

(6) x=Ψϑ

where **ϑ** is the associated coefficient vector and the number of nonzero in **ϑ** is *K* with *K* ≪ *N*. The recently emerged CS theory indicates that such a signal can be exactly recovered with high probability from *M* (*M* < *N*) linear measurements, and the related observation procedure can be expressed as

(7) y=Φx=ΦΨϑ=Θϑ

where **Φ** is an *M* × *N* measurement matrix and **y** is the measurement vector of length *M*. Since *M* < *N*, solving Equation (7) is highly underdetermined. CS provides a feasible way to solve this problem by exploiting the sparsity of the signal. According to [1,2], provided that the number of measurements satisfies *M*=*O*(*K* log(*N*/*K*)) CS is capable of recovering the sparse signal **x** (via its coefficient vector **ϑ**) from the measurements **y** by solving the following constrained optimization problem:

(8) $$\begin{array}{ccc} \underset{\vartheta }{\min}{\left\|\vartheta \right\|}_{1} & s.t. & y=\Theta \vartheta \end{array}$$

Where ‖**ϑ**‖ is the *l*_1_ norm of the vector **ϑ**. The minimization problem of Equation (8) is often referred to as Basis Pursuit (BP), which can be solved by linear programming.

### 3.2. CS-SAR Imaging Approach

To apply the CS framework into the SAR imaging, the random undersampling scheme is implemented in both range and azimuth dimensions firstly. Specifically, a pseudorandom sequence {*c_l_*, *l* = 1, 2, ⋯, *L*} is randomly generated for undersampling in azimuth. Note that for the case when *c_l_*=1, temporal samplers are available for SAR imaging, while for *c_l_* = 0, the samplers are discarded. For each *c_l_*=1, a similar pseudorandom sequence {*c_ls_*, *s* = 1, 2, ⋯, *S*} is produced to reduce the sampler number in range. Obviously for any two indices in {*c_l_*}, the corresponding range sampling sequence {*c_ls_*} varies randomly. To reduce the sampling number, the numbers of the nonzero elements in {*c_l_*} and {*c_ls_*} are much smaller than *L* and *S*. By representing the pseudorandom sequences in matrix form, we have

(9) $$\Phi =\text{diag}\left\{c_{1}\text{diag}\left[\left\{c_{1s}\right\}\right],c_{2}\text{diag}\left[\left\{c_{2s}\right\}\right],\cdots ,c_{L}\text{diag}\left[\left\{c_{Ls}\right\}\right]\right\}$$

With **Φ**, some of the samplers for SAR imaging are discarded, which reduces the sampling rate. By incorporating the sparsity constraint on the scene ***q***, SAR imaging can be transformed into the following minimization problem

(10) $$\begin{array}{ccc} \hat{\text{q}}=\arg\underset{\text{q}}{\min}{\left\|\text{q}\right\|}_{1}, & s.t. & \left\{ \begin{array}{l} \text{y}=\Phi \text{Cq}+\varepsilon \\ \text{q}=\Psi \vartheta \end{array}\right. \end{array}$$

Obviously, SAR imaging quality largely depends on the space **Ψ**. When **Ψ** is an identity matrix, the point-like features of the unknown scene can be enhanced. When **Ψ** is a fixed basis, such as DCT or wavelet, different features of the scene can be enhanced [9]. Choosing a fixed and known basis is appealing due to its easy implementation. However, in most cases, local structure features vary across the scene and usually appear irregularly. For instance, different segments of a SAR image have different waveforms, and such varying second-order statistics exhibit varied sparsity in spatial domain. In such cases, the above fixed bases lack adaptivity to characterize the varied image structures and will suffer from limited representation of the signal.

## 4. ASR-CS-SAR Approach

In this section, by exploring the structural similarity among pixels, a weighted AR model is obtained. Based on it, a novel compressive SAR imaging approach with adaptive sparse representation is proposed. Finally, the computational complexity of the proposed approach is discussed.

### 4.1. AR Model and Image Structural Similarity

Based on the fact that an image is a non-stationary random Markov field of a modest order, the AR model [11] has shown to be effective in depicting local image structure. Specifically, the pixel ***q****_i_* can be represented as

(11) $${\text{q}}_{i}=\sum_{iοk\in \Omega_{i}}\zeta_{i}{\text{q}}_{iοk}+\omega_{i}$$

where **Ω***_i_* is a local neighborhood around the pixel ***q****_i_*, **ζ***_i_* stands for the associated AR parameter, and **ω***_i_* is a random perturbation independent of spatial location and the image pixel. The subscript *iok* denotes the *k-th* neighbor of pixel ***q****_i_* in the **Ω***_i_* stored in a raster scan. Generally speaking, using a large **Ω** is more efficient to characterize image local waveform. However, considering that the AR model is based on the local stationary assumption, using a large **Ω** may lead to an over-fitting problem. Thus an empirical 3 × 3 local neighbor is used here to achieve the optimum balance between efficiency and robustness. Normally, the calculation of **ζ***_i_* is implemented in a local window ***W****_i_* where the statistical stationary assumption holds. Therefore, by adjusting **ζ***_i_* to fit the variant local waveform, the local image structure can be illustrated by spatial contiguous pixels.

**Figure 1.**
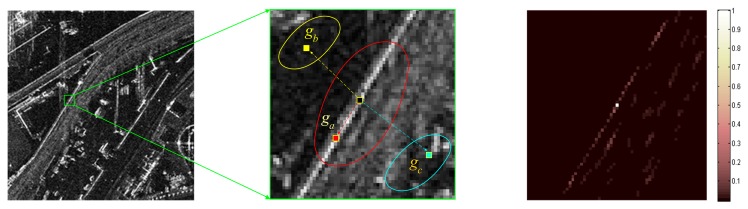
(**a**) SAR image; (**b**) non-stationary patch; (**c**) distribution of the SSP.

The AR model performs well in a local stationary window. However, in practice, the waveforms vary significantly across the whole SAR image, and the statistical stationary assumption does not always hold [13]. Especially when the scale of a local structure feature is smaller than the selected local window size, the piecewise stationary assumption is violated. Take Figure 1 for example, where Figure 1b is the close-up view of the selected local area in the green window in Figure 1a. Although the statistical property keeps stationary within each ellipse region, it varies significantly across various ellipses. To be specific, to estimate the pixel ***q****_i_* on the edge, samples within the red region (e.g., pixel ***q****_a_*) can provide more reasonable local structure information than those in the yellow (e.g., pixel ***q****_b_*) and blue (e.g., pixel ***q****_c_*) regions. If pixels in the yellow and blue regions are engaged with the same weights, using the AR model in Equation (11) will lead to a degraded description of the local image structure.

Considering that any two pixels share similarity, we introduce the structural similarity probability (SSP) as weight into the AR model to further promote its capability of characterizing the local image structure. Specifically, to achieve an adaptive representation of the pixel *q_i_* in Figure 1, we prefer to impose large weight on the pixel that shares a similar local structure (e.g., *q_a_*), while small weight or nearly zero on the pixel that has large disparity (e.g., pixels *q_b_* and *q_c_*). Mathematically, the SSP between any two pixels *q_i_* and *q_j_* is quantitatively evaluated by the local structure vector *Z_i_* and *Z_j_*, where *Z_i_* denotes a square 8-connected neighborhood centered at pixel *q_i_*. To be specific, the SSP between pixels *q_i_* and *q_j_* is modeled as the Gaussian function of the Euclidean distance between their local structure vectors:

(12) $$\chi_{ij}=\exp\left(-{\left\|{\vec{\text{Z}}}_{i}-{\vec{\text{Z}}}_{j}\right\|}_{2}^{2}/h^{2}\right)$$

where *h* acts as a degree of filtering that controls the shape of the exponential function. To make the structural similarity independent of the image intensity field, the normalized ***Z⃗***_*i*_=(***Z****_i_*+η)/(*max*(***Z****_i_*)+η) is presented, in which *max*(·)returns the maximum value. For more details, one can refer to [14]. To facilitate understanding, the SSP between any pixel and the center pixel ***q****_i_* in the local window Figure 1b is calculated based on Equation (12) and the associated result is shown in Figure 1c, where the darker intensity grid means significantly lower weight, and *vice versa*. It is evident that the weights along the edge are nonzero while most of the others in the local window are zero or nearly zero, which indicates that the pixels along the edge can provide more efficient local structure information compared with other pixels. Thus, the profile characterized by the SSP distribution is consistent with the local image structure.

### 4.2. Adaptive Sparse Representation Scheme

**Figure 2.**
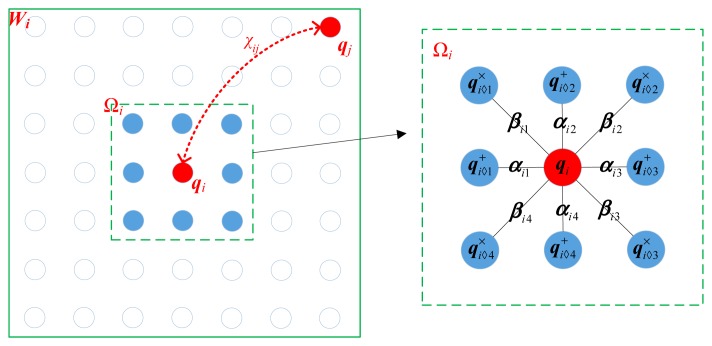
The spatial configuration of the weighted AR model.

To circumvent the risk of data overfitting, the AR model in Equation (11) is normally split into two lower order models as

(13) $$\begin{array}{l} {\text{q}}_{i}=\underset{1\leq m\leq 4}{\text{∑}}\alpha_{i⋄m}{\text{q}}_{i⋄m}^{+}+\omega_{i}^{+}\\ {\text{q}}_{i}=\underset{1\leq m\leq 4}{\text{∑}}\beta_{i⋄m}{\text{q}}_{i⋄m}^{\times }+\omega_{i}^{\times } \end{array}$$

where ${\text{q}}_{i⋄m}^{+}$ and ${\text{q}}_{i⋄m}^{\times }$ refer to *m*-th neighbors of the pixel ***q****_i_* in the axial and diagonal directions, respectively, as shown in Figure 2. Naturally, the associated parameter **ζ***_i_* in Equation (11) reduces to two sets of parameters **α***_i_* and **β***_i_*, where **α***_i_*={*α_i_*_◊_*_m_*,*m*=1,2,3,4} and **β***_i_*={*β_i_*_◊_*_m_*,*m*=1,2,3,4} characterize the correlation in the axial and diagonal directions, respectively.

Remembering that similarity among pixels can be used to further promote the capability of describing the structural property, we incorporate the SSP distribution into the AR model, then a weighted AR model can be obtained. Specifically, for a given local window *W_i_*, the parameters **α***_i_* and **β***_i_* corresponding to the center pixel ***q****_i_* can be estimated via

(14) $$\begin{array}{l} {\hat{\alpha }}_{i}=\arg\underset{\alpha_{i}}{\min}\underset{j\in W_{i}}{\text{∑}}\chi_{ij}{\left({\text{q}}_{j}-\underset{1\leq m\leq 4}{\text{∑}}\alpha_{i⋄m}{\text{q}}_{j⋄m}^{+}\right)}^{2}\\ {\hat{\beta }}_{i}=\arg\underset{\beta_{i}}{\min}\underset{j\in W_{i}}{\text{∑}}\chi_{ij}{\left({\text{q}}_{j}-\underset{1\leq m\leq 4}{\text{∑}}\beta_{i⋄m}{\text{q}}_{j⋄m}^{\times }\right)}^{2} \end{array}$$

where χ*_ij_* is calculated by Equation (12). When **α***_i_* and **β***_i_* are determined, each pixel ***q****_i_* can be formulated as

(15) $$\begin{array}{l} {\text{q}}_{i}={\text{d}}_{i}\text{q}+\omega_{i}\\ d_{i,j}=\left\{ \begin{array}{ll} \alpha_{i⋄m}, & \text{if}\;{\text{q}}_{j}\;\text{is the}m-\text{th axial neighbor of}\;{\text{q}}_{i}\\ \beta_{i⋄m}, & \text{if}\;{\text{q}}_{j}\;\text{is the}\;-\text{th diagonal neighbor of}\;{\text{q}}_{i}\\ 0, & \;\text{others} \end{array}\right. \end{array}$$

where ***d****_i_* is a vector of length *N* × 1 and the matrix form of Equation (15) can be expressed as

(16) q=Dq+ω

where ***D***=[***d***_1_,***d***_2_,⋯***d****_N_*]*^T^* and ***ω*** accounts for the residual. As ***d****_i_* is determined by the AR parameters, and only 8 entries are nonzero at most, ***d****_i_* is a sparse vector.

### 4.3. The Proposed Approach

By imposing the sparsity constraint on each ***d****_i_*, a novel compressive SAR image framework with adaptive sparse representation (ASR-CS-SAR for short) can be obtained as

(17) $$\underset{D,\text{q}}{\min}\sum_{i}{\left\|d_{i}\right\|}_{1},s.t.\left\{ \begin{array}{l} \text{y}=\Phi \text{Cq}+\varepsilon \\ \text{q}=\text{Dq}+\omega \end{array}\right.$$

As the structure described by ***D*** is intrinsic to the real physical one, the sparse representation derived from the modified AR model is more adaptive than that with a fixed basis. Though the problem in Equation (17) is convex, such a combinational optimization problem still cannot be easily solved since a better estimation of ***D*** returns a more accurate representation of the SAR image, which in turn leads to a smaller residual ***ω***. Motivated by this, we replace the regularized term $\underset{i}{\text{∑}}{\left\|{\text{d}}_{i}\right\|}_{1}$ with the sparsity constraint on ***ω***, and then a relaxed version of Equation (17) can be obtained as

(18) $$\underset{D,\omega ,\text{q}}{\min}\sum_{i}{\left\|\omega_{i}\right\|}_{1},\;s.t.\left\{ \begin{array}{l} \text{y}=\Phi \text{Cq}+\varepsilon \\ \omega =\left(\text{I}-\text{D}\right)\text{q} \end{array}\right.$$

Since ***D*** greatly depends on the prior information of the image, SAR imaging as well as the estimation of ***D*** should be jointly optimized. Thus, an optimization approach for iterative SAR imaging and updating of ***D*** is proposed. Specifically, for a given image ***q***, ***D*** can be calculated according to Equations (14) and (15). When given ***D***, Equation (18) reduces to a conventional SAR imaging problem. For convenience of expression, let ***A***=***I***−***D***, and the Augmented Lagrange Multiplier (ALM) technique is adopted to convert the constrained minimization problem Equation (18) into an unconstrained optimization problem

(19) $$\begin{array}{l} \left(\hat{\text{q}},\hat{\omega }\right)=\arg\underset{\text{q},\omega ,\text{A}}{\min}L\left(\text{q},\omega ,\text{A}\right)\\ L\left(\text{q},\omega ,\text{A}\right)={\left\|\omega \right\|}_{1}-\lambda^{T}(\text{Aq}-\omega )+\frac{\nu }{2}{\left\|\text{Aq}-\omega \right\|}_{2}^{2}+\frac{\tau }{2}{\left\|\text{y}-\Phi \text{Cq}\right\|}_{2}^{2} \end{array}$$

where λ is the Lagrangian multiplier, and *ν* and τ are positive scalar parameters. Since ***q*** and ***ω*** are unknown, the above minimization problem can be solved by alternatively optimizing the following sub-problems, *i.e.*,

(20) $$\hat{\omega }=\arg\underset{\omega }{\min}\left\{{\left\|\omega \right\|}_{1}+\lambda^{T}\omega +\frac{\nu }{2}{\left\|\text{Aq}-\omega \right\|}_{2}^{2}\right\}$$

(21) $$\hat{\text{q}}=\arg\underset{\text{q}}{\min}\left\{-\lambda^{T}\text{Aq}+\frac{\nu }{2}{\left\|\text{Aq}-\omega \right\|}_{2}^{2}+\frac{\tau }{2}{\left\|\text{y}-\Phi \text{Cq}\right\|}_{2}^{2}\right\}$$

Clearly, the minimizing of the sub-problems has a close-form formula and thus can be solved effectively. For a fixed image ***q***, we take the gradient of Equation (20) with respect to ***ω***, and yield the following shrinkage formula

(22) $$\hat{\omega }=\max\left\{{\left\|\kappa \right\|}_{1}-\frac{1}{\beta },0\right\}\frac{\kappa }{{\left\|\kappa \right\|}_{1}}$$

where κ=***Aq***−*λ*/*v*, while for a fixed ***ω***, Equation (21) is a quadratic minimization problem and ***q*** can be given by

(23) $$\hat{\text{q}}={\left[\tau {\left(\Phi \text{C}\right)}^{T}\Phi \text{C}+\nu {\text{A}}^{T}\text{A}\right]}^{-1}\times \left[\tau {\left(\Phi \text{C}\right)}^{T}y+\nu {\text{A}}^{T}\omega +{\text{A}}^{T}\lambda \right]$$

Due to the large computation of Equation (23), in our implementation, conjugate gradient (CG) algorithm is adopted to estimate ***q***. During the iterative process, parameters including λ, *ν*and τ should be updated and the detailed updated formulas at *l*-th iteration can be expressed as [15,16]

(24) $$\begin{array}{l} \lambda^{\left(l\right)}=\lambda^{\left(l-1\right)}-\nu^{\left(l-1\right)}\left({\text{Aq}}^{\left(l\right)}-\omega^{\left(l\right)}\right)\\ \tau^{\left(l\right)}=\varsigma \tau^{\left(l-1\right)}\\ \nu^{\left(l\right)}=\varsigma \nu^{\left(l-1\right)} \end{array}$$

where ς is a constant. In general, the reconstruction of the image ***q*** is implemented iteratively. The initial image is estimated roughly, then the estimated one is used to update ***ω*** and ***D***, which are in turn used to improve the reconstruction of ***q***. Such an iterative minimization process terminates until the stopping criterion is satisfied. Specifically, the overall algorithm of the proposed ASR-CS-SAR approach is outlined in Table 1.

### 4.4. Computational Complexity

The computational complexity of the proposed approach mainly stems from three parts: the initial reconstruction, the estimation of the weighted AR parameters, and SAR imaging with the adaptive sparse representation. First, a BP algorithm is applied to solve Equation (10) for an initial image, which requires *O*(*N*log*N*) operations. Next, the computational complexity in the second part includes the calculation of SSP among pixels and the estimation of **α** and **β**. The former is solved by Equation (12), which needs *O*(*NU*)operations, where *U* is the order of the AR model, while the latter is obtained by solving a least square problem in Equation (14) thatneeds *O*(*NWU*^2^) operations, where $\sqrt{W}$ is the width of the local window. Thus total *O*(*NU* + *NWU^2^*) operations are needed in this part. Finally, SAR imaging with the adaptive sparse representation refers to two steps: updating **ω** according to Equation (22), and updating ***q*** according to Equation (23). The former requires *O*(*N^2^*)operations, while directly solving Equation (23) requires *O*(*N*^3^ + *MN*^2^ + *MN*)operations. In our manuscript, a conjugate gradient (CG) algorithm is adopted to compute Equation (23) to reduce the computer complexity. In fact, matrix-vector multiplication is the dominating operation in the CG algorithm, which can be executed in parallel to speed up the algorithm by taking advantage of the multi-core CPU and GPU structure. In addition, the multiplication ***A****^T^****A*** can be implemented by fast FFT.As [17] mentioned, if the CG solver converges in *k* iterations, then the associated computation complexity can be reduced to *O*(4*Nk*). Thus the cost of SAR imaging with adaptive sparse representation is *O*(*N_w_*(4*Nk* + *N^2^*)), where *N_w_* denotes the whole iteration number.

## 5. Experiments

In this section, experiments over various scenarios are carried out to demonstrate the effectiveness of the proposed approach. In Section 5.1, a visual comparison is conducted between synthetic SAR data. In Section 5.2, quantitative comparisons between our approach and the conventional one using fixed sparse spaces are conducted. In Section 5.3, the application of the proposed approach in real SAR is given.

In our approach, the window size for the estimation of the weighted AR model is empirically set as 11 × 11. During these experiments, the proposed approach is compared with conventional CS-SAR imaging approaches with sparse representation in wavelet [9], DCT [10], and TV [4]. Furthermore, two image quality metrics, including the peak signal to noise ratio (PSNR) and structural similarity (SSIM) [18], are introduced for image quality evaluation, defined as follows:

(a) Peak signal-to-noise ratio (PSNR) stands for the ratio between the maximum possible power of a signal and the power of corrupting noise that affects the fidelity of its representation:
  (25) $$\begin{array}{l} \text{MSE}=\frac{1}{MN}\overset{N}{\underset{i=0}{\text{∑}}}\overset{M}{\underset{j=0}{\text{∑}}}{\left[\text{q}\left(i,j\right)-\hat{\text{q}}\left(i,j\right)\right]}^{2}\\ \text{PSNR}=-10\log_{10}\left(\frac{\text{MSE}}{\rho_{\max}}\right) \end{array}$$
  where ***q*** and ***q̂*** denote the original image and the reconstructed image, respectively. *MN* is the total number of pixels in***q***. ρ*_max_* is the maximum energy of the noisyimage.
(b) Structural Similarity (SSIM) is a metric for measuring the similarity between two images, different from PSNR, which is dependent on the average luminance and contrast only. The structural information in an image is defined as those attributes representing an object's structures within the scene; this metric has been proven to be inconsistent with human eye perception. Mathematically, SSIM can be expressed as
  (26) $$\text{SSIM}=\frac{\left(2\mu_{\text{q}}\mu_{\hat{\text{q}}}+V_{1}\right)\left(2\sigma_{\text{q}\hat{\text{q}}}+V_{2}\right)}{\left(\mu_{\text{q}}^{2}\mu_{\hat{\text{q}}}^{2}+V_{1}\right)\left(2\sigma_{\text{q}}^{2}\sigma_{\hat{\text{q}}}^{2}+V_{2}\right)}$$
  where μ*_q_* and μ_*q̂*_ represent the mean of the image ***q*** and ***q̂***, respectively. $\sigma_{\text{q}}^{2}$ and $\sigma_{\text{q}}^{2}$ stand for the corresponding variance. σ_*qq̂*_ is the covariance between ***q*** and ***q̂***. *V*_1_ and *V*_2_ are two variables to stabilize the division with weak denominator. The range of values for SSIM lies between 1 and −1. Note that a higher SSIM value corresponds to a better restoration, and *vice versa*.

### 5.1. Visual Comparison

**Figure 3.**
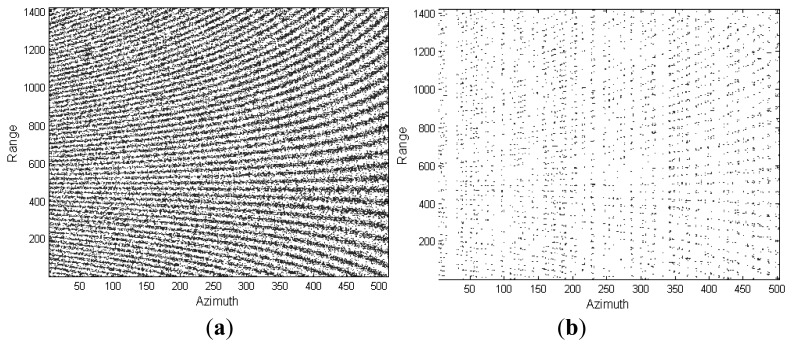
(**a**) Full SAR echo; (**b**) Undersampled return.

**Figure 4.**
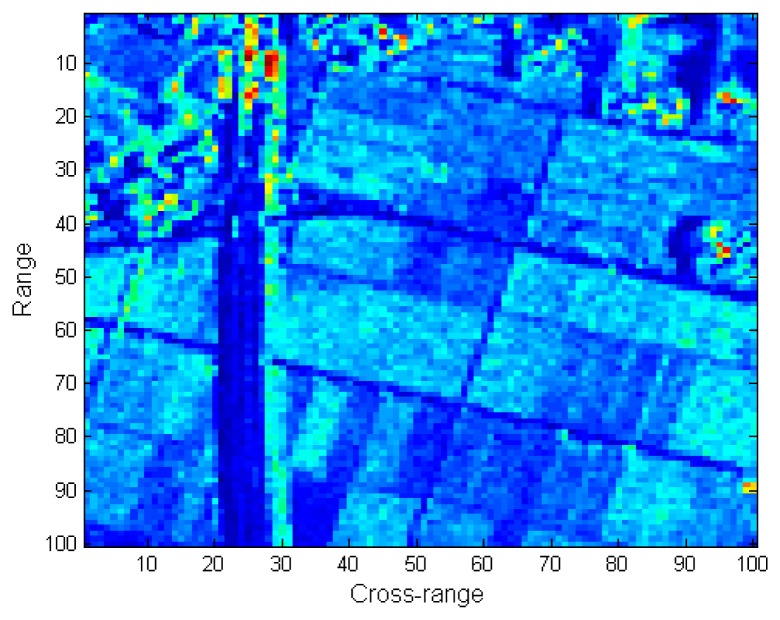
Image recovered from full data.

In this experiment, a visual comparison is presented to show the feasibility of the proposed ASR-CS-SAR imaging approach. The primary radar parameters are enumerated in Table 2. By applying the random undersampling scheme mentioned in Section 2, 33% of Nyquist rate samples in range as well as 25% in azimuth are adopted, which implies that only 1/12 full aperture data is used for reconstruction. Figure 3a,b depict the full echo data and the associated undersampled return, respectively. To facilitate comparison, SAR imaging with full aperture data is displayed in Figure 4.

**Figure 5.**
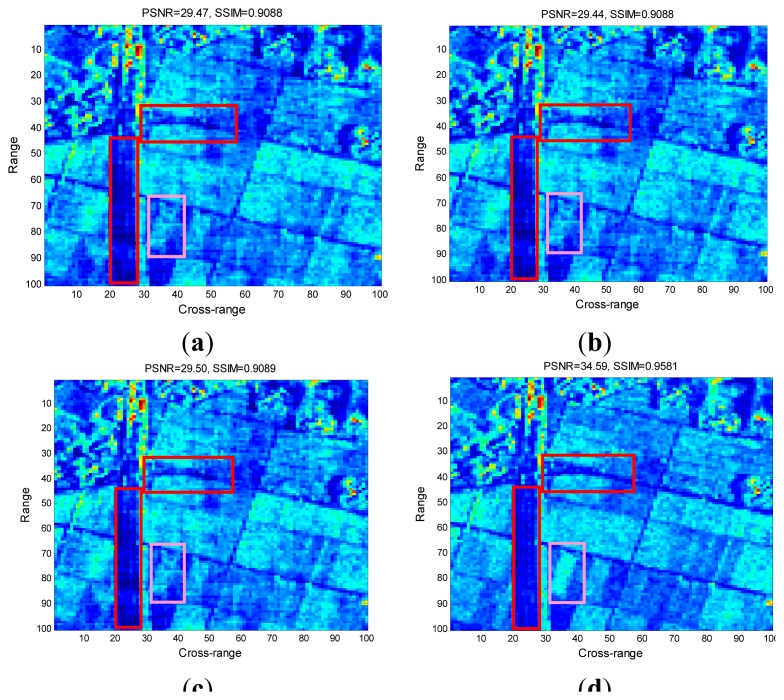
Reconstruction comparison from undersampled SAR echoes. (**a**) Imaging by [9]; (**b**) Imaging by [10]; (**c**) Imagingby [4]; (**d**) Imaging by ASR-CS-SAR.

Figure 5a–c present the reconstructed images by the conventional CS-SAR approaches, and Figure 5d is the image reconstructed by the proposed ASR-CS-SAR approach. From these figures, we can see that most information on the SAR images is preserved. However, for the conventional CS-SAR approaches, they still suffer from a series of artifacts. Specifically, the textures of the roads in the red rectangle window are seriously merged and it is hard distinguish them from the terraced fields. Similarly, the terrace in the pink rectangle window has been blurred. However, those artifacts are suppressed in Figure 5d and a sharper and clearer visual result can be obtained by the proposed approach. The superior performance of the ASR-CS-SAR is also demonstrated by the values of image quality metrics, *i.e.*, PSNR and SSIM, for the maximum gap in PSNR between our approach and the competing methods is up to 7.15 dB.

**Figure 6.**
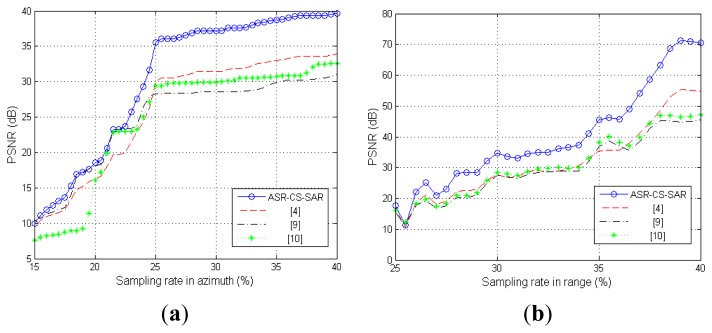
Comparison of the reconstruction performance. (**a**)33% sampling rate in range; (**b**) 25% sampling rate in azimuth.

To further test the stability of the proposed algorithm, we change the sampling rates in azimuth or range with another fixed, and compare the images reconstructed by our proposed algorithm and other algorithms. Figure 6 presents the comparison results.

Figure 6a shows that all the PSNR curves rise sharply as the sampling rate in azimuth increases. However, our proposed approach outperforms others for each sampling rate; furthermore, the advantage becomes more and more apparent with the increase of the sampling rate. Similar conclusions can also be drawn from the results in Figure 6b.

### 5.2. Quantitative Comparison and Universality Analysis

**Figure 7.**
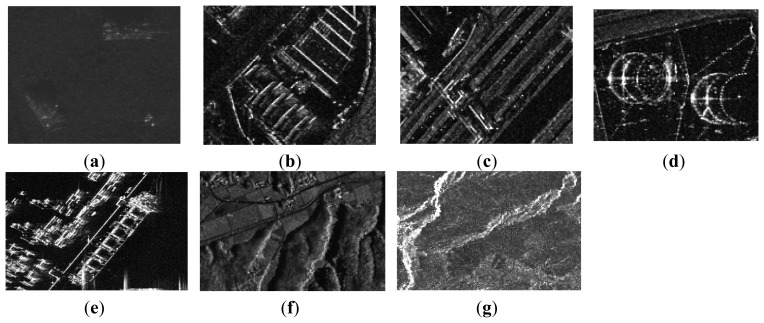
Test images. (**a**) Image 1; (**b**) Image 2; (**c**) Image 3; (**d**) Image 4; (**e**) Image 5; (**f**) Image 6; (**g**) Image 7.

To demonstrate the universality of the proposed approach, simulations over a variety of SAR images under different sampling rates (including a low sampling mode and a high sampling mode) were carried out. More specifically, in the low sampling mode, 33% of Nyquist rate samples in range and 25% in azimuth were adopted, while in the high sampling mode, the sampling numbers in range and azimuth increased to 38% and 30%, respectively. All the other radar parameters were the same as in the first experiment. In Figure 7, we list all the SAR images.

Table 3 illustrates the quantitative performance metrics, including PSNR and SSIM, *vs.* the number of samples for different CS SAR imaging approaches.

It turns out that, for all test images, the proposed approach significantly outperforms other competing approaches in PSNR and SSIM. Especially for the images with rich texture and sharp edges (e.g., mountains and terraced fields), the average gain of proposed approach over other competing approaches can be up to 4 dB in PSNR and 0.02 in SSIM for the low sampling mode. When the sampling number increases, the superiority is enlarged for most complex images. The superiority is mainly due to the capability of the adaptive sparse representation in exploiting the inherent structure of the image. In conclusion, all these quantitative results manifest the fact that the proposed approach is highly competitive and applicable to various types of SAR images.

### 5.3. Application to Real SAR Data

**Figure 8.**
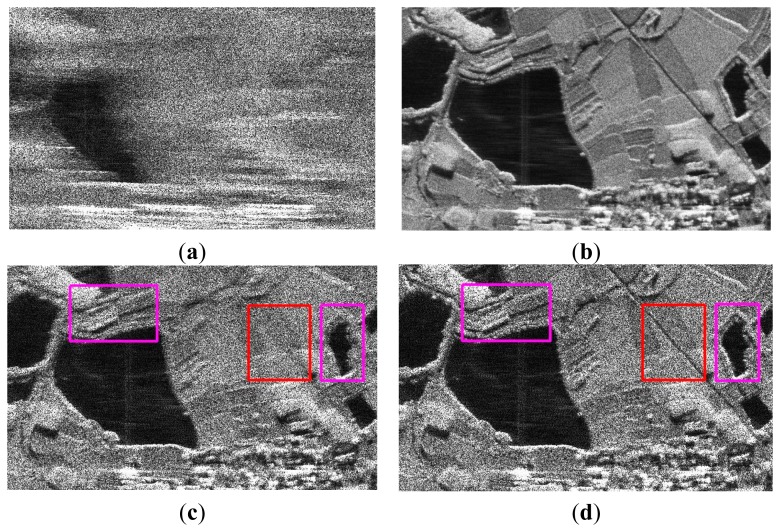
Comparison of the reconstructed images: (**a**) The defocus image; (**b**) RD imaging with motion compensation; (**c**) CS-SAR imaging with motion compensation [18]; and (**d**) ASR-CS-SAR imaging with motion compensation.

In this experiment, the proposed ASR-CS-SAR imaging approach is applied to real SAR data. The data were acquired by an airborne SAR, and the associated radar parameters are listed in Table 4. In practice, due to the slight deviation of the platform from the nominal trajectory, the inaccuracy of the observation model caused by the additional uncertain factor results in inevitable phase error in the SAR data. Hence, the conventional reconstruction of SAR images suffers from degradation due to the phase error. As shown in Figure 8a, the defocused SAR image has been blurred and is hard to distinguish. To achieve a well-focused SAR image, the unknown phase error should be compensated for first. In our experiment, we adopt the strategy in [19], where the phase error correction and the compressive SAR imaging are iteratively operated. In this strategy, the imaging quality directly influences the estimation accuracy of the phase error, which in turn affects the image reconstruction. To be fair, the same autofocus scheme is carried out in our proposed approach. In this experiment, SAR echoes are undersampled in the azimuth dimension, and only 30% of the original raw data in azimuth is available. For better comparison, the perfectly focused image recovered from full echo data is shown in Figure 8b.

The images reconstructed by the CS-SAR imaging approach with motion compensation [19] and our proposed approach are shown in Figure 8c,d, respectively. Compared with the focused image in Figure 8b, most of the image contents have been preserved in Figure 8c, while some regions are still defocused. Specifically, the road marked in the red rectangle has nearly disappeared. In addition, the structure of the target as well as the edge of the lake (in the pink rectangles) is also blurred with the surrounding region. In contrast, all these problems are solved by our proposed approach, and a clearer and sharper image is obtained in Figure 8d. This is due to the fact that a more precise image can be reconstructed by the proposed approach, and thus the unknown phase error can be removed more completely.

## 6. Conclusions

By integrating similarity among pixels into the AR model, we propose a weighted AR model to exploit the structural sparsity in a local non-stationary window. A novel compressive SAR imaging approach with adaptive sparse representation was developed. Experimental results over synthetic and real SAR echo data demonstrated the effectiveness and universality of the proposed approach.

## Figures and Tables

**Table 1.** The proposed ASR-CS-SAR approach.

1.	Initialization:
	(a) Compute an initial image ***q***^(0)^ by solving Equation (10) with the wavelet basis selected;
	(b) Suppose *l* = 1, ω^(0)^ = 0, λ^(0)^ = 1.5, δ_e_ = 10^−6^, and let *I_max_* be the maximum iterations;
2.	Updating the parameter matrix ***D***^(^*^l^*^)^
	(a) Compute the SSR of the image according to Equation (12);
	(b) Update the matrix ***D***^(^*^l^*^)^ according to Equations (14) and (16);
3.	SAR imaging with fixed ***A***^(^*^l^*^)^ = ***I*** − ***D***^(^*^l^*^)^
	(a) Update ***ω***^(^*^l^*^)^ according to Equation (22) with fixed ***q***^(^*^l^*^)^;
	(b) Update ***q***^(^*^l^*^)^ according to Equation (23) with fixed ***ω***^(^*^l^*^)^;
	(c) Update the related parameters according to Equation (24);
4.	If, ${{\left\|{\text{q}}^{\left(l\right)}-{\text{q}}^{\left(l-1\right)}\right\|}_{2}^{2}/\left\|{\text{q}}^{\left(l-1\right)}\right\|}_{2}^{2}<\delta_{e}$ or *l* ≥ *I_max_* output the image ***q***^(^*^l^*^)^; otherwise let *l* = *l* + 1 and jump to 2.

**Table 2.** Radar Parameters.

**Center Frequency**	**12 GHz**
Pulse width	30 μs
Bandwidth	350 MHz
Coherent accumulation angle	2.3°
Signal-to-noise-ratio	15 dB

**Table 3.** Quality metrics for reconstructed images.

**Image**	**Method**	**PSNR**		**SSIM**	

		**Low-Sampling Mode**	**High-Sampling Mode**	**Low-Sampling Mode**	**High-Sampling Mode**
Image 1	[9]	29.96	33.72	0.8463	0.9516
	[10]	30.39	33.74	0.8633	0.9517
	[4]	30.43	34.94	0.8643	0.9568
	ASR-CS-SAR	34.07	37.97	0.9491	0.9734

Image 2	[9]	29.94	31.48	0.9495	0.9605
	[10]	29.91	31.35	0.9500	0.9612
	[4]	29.92	31.72	0.9494	0.9625
	ASR-CS-SAR	32.50	35.28	0.9701	0.9820

Image 3	[9]	32.45	36.69	0.9766	0.9905
	[10]	32.49	36.71	0.9766	0.9905
	[4]	32.53	36.74	0.9769	0.9907
	ASR-CS-SAR	35.02	39.95	0.9845	0.9926

Image 4	[9]	28.51	36.67	0.9810	0.9961
	[10]	28.77	36.70	0.9817	0.9961
	[4]	28.96	36.74	0.9821	0.9961
	ASR-CS-SAR	32.77	40.01	0.9922	0.9984

Image 5	[9]	26.27	31.48	0.9738	0.9887
	[10]	26.28	31.53	0.9739	0.9984
	[4]	26.30	31.59	0.9743	0.9887
	ASR-CS-SAR	31.34	36.03	0.9906	0.9953

Image 6	[9]	26.46	33.06	0.9370	0.9722
	[10]	26.63	33.08	0.9379	0.9723
	[4]	26.86	33.13	0.9395	0.9724
	ASR-CS-SAR	31.86	38.43	0.9634	0.9920

Image 7	[9]	28.87	34.08	0.9396	0.9895
	[10]	28.89	34.21	0.9370	0.9899
	[4]	28.93	34.28	0.9379	0.9900
	ASR-CS-SAR	33.89	40.26	0.9436	0.9965

**Table 4.** Radar Parameters.

**Center Frequency**	**9.55 GHz**
Pulse width	15 μs
Bandwidth	508 MHz
Pulse Repetition Frequency	1000 Hz
Δθ	2.5°
